# Biomarkers Associated With Aortic Valve Calcification: Should We Focus on Sex Specific Processes?

**DOI:** 10.3389/fcell.2020.00604

**Published:** 2020-07-10

**Authors:** Frederique E. C. M. Peeters, Elton A. M. P. Dudink, Bob Weijs, Larissa Fabritz, Winnie Chua, Bas L. J. H. Kietselaer, Joachim E. Wildberger, Steven J. R. Meex, Paulus Kirchhof, Harry J. G. M. Crijns, Leon J. Schurgers

**Affiliations:** ^1^Department of Cardiology and CARIM, Maastricht University Medical Center+, School for Cardiovascular Diseases, Maastricht, Netherlands; ^2^Institute of Cardiovascular Sciences, University of Birmingham, Birmingham, United Kingdom; ^3^Department of Radiology and Nuclear Medicine and CARIM, Maastricht University Medical Center+, School for Cardiovascular Diseases, Maastricht, Netherlands; ^4^Department of Clinical Chemistry and CARIM, Maastricht University Medical Center+, School for Cardiovascular Diseases, Maastricht, Netherlands; ^5^Department of Biochemistry and CARIM, Maastricht University, School for Cardiovascular Diseases, Maastricht, Netherlands

**Keywords:** aortic valve calcification, biomarkers, sex-specific, fibrosis, inflammation

## Abstract

**Objective:**

Circulating biomarkers are useful in detection and monitoring of cardiovascular diseases. However, their role in aortic valve disease is unclear. Mechanisms are rapidly elucidated and sex differences are suggested to be involved. Therefore, we sought to identify biomarkers involved in aortic valve calcification (AVC) stratified by sex.

**Methods:**

Blood samples of 34 patients with AVC (without further overt cardiovascular disease, including absence of hemodynamic consequences of valvular calcification) were compared with 136 patients without AVC. AVC was determined using computed tomography calcium scoring. Circulating biomarkers were quantified using a novel antibody-based method (Olink Proseek Multiplex Cardiovascular Panel I) and 92 biomarkers were compared between patients with and without AVC.

**Results:**

In the overall population, Interleukin-1 Receptor Antagonist and pappalysin-1 were associated with increased and decreased odds of having AVC. These differences were driven by the male population [IL1RA: OR 2.79 (1.16–6.70), *p* = 0.022; PAPPA: OR 0.30 (0.11–0.84), *p* = 0.021]. Furthermore, TNF-related activation-induced cytokine (TRANCE) and fibroblast growth factor-23 were associated decreased odds of having AVC, and monocyte chemotactic protein-1 was associated with increased odds of having AVC [TRANCE: OR 0.32 (0.12–0.80), *p* = 0.015; FGF23: OR 0.41 (0.170–0.991), *p* = 0.048; MCP1: OR 2.64 (1.02–6.81), *p* = 0.045]. In contrast, galanin peptides and ST2 were associated with increased odds of having AVC in females [GAL: OR 12.38 (1.31–116.7), *p* = 0.028; ST2: OR13.64 (1.21–153.33), *p* = 0.034].

**Conclusion:**

In this exploratory study, we identified biomarkers involved in inflammation, fibrosis and calcification which may be associated with having AVC. Biomarkers involved in fibrosis may show higher expression in females, whilst biomarkers involved in inflammation and calcification could associate with AVC in males.

## Introduction

Aortic valve calcification (AVC) is a major determinant in leaflet stiffening and progression of aortic valve disease. AVC, also known as calcific aortic valve disease (CAVD), calcific aortic valve stenosis (CAVS), or aortic valve stenosis (AS), is a spectrum of disease, ranging from aortic valve sclerosis to severe AS. Aortic valve sclerosis is defined as diffuse thickening of the aortic valve without significant blood flow obstruction. The occurrence of aortic valve sclerosis is common, even in relatively young populations: its incidence increases from 1.9 to 8.8% with increasing age and its prevalence is ∼40% in patients >75 years. Moreover, it is associated with increased cardiovascular risk (Coffey et al., 2014, 2016). Over time, aortic valve disease progresses slowly, and ∼2% of patients develop hemodynamically significant AS per year (Messika-Zeitoun et al., 2007; Novaro et al., 2007). Aortic valve stenosis is defined as narrowing of the valve causing blood flow obstruction.

With an increasingly elderly population, disease burden of aortic valve stenosis will increase in the coming years. Due to the complexity, challenges, and cost of management, the number of patients with an indication for treatment is expected to double by 2050 in Europe and the United States (Osnabrugge et al., 2013). Whereas aortic valve stenosis was considered a passive disease whereby by “wear and tear” resulted in calcification of the valve, emerging evidence showed that it is an active disease, involving highly complex and tightly regulated pathways (Rajamannan et al., 2011; Pawade et al., 2015). However, we still lack precise molecular insight in pathophysiological processes and their exact contribution to aortic valve stenosis and its progression.

Pathophysiological mechanisms involved in the initiation and progression of aortic valve disease are being rapidly elucidated, but their exact contribution and extent of their involvement remain to be investigated (Dweck et al., 2013). Whereas aortic valve disease used to be considered a passive and degenerative process, it is now appreciated to be an active process with involvement of multiple cellular and molecular pathways in inflammation, fibrosis and calcification. Calcification is one of the critical processes in AS progression (Otto et al., 1994; Carabello and Paulus, 2009; Pawade et al., 2015; Peeters et al., 2018). Sex specificity of processes involved are suggested to be present in aortic valve disease, yet are largely unresolved (Sritharen et al., 2017). The extent of contribution of calcification as well as fibrosis to the progression of AS is a matter of debate and seems related to sex differences (Hervault and Clavel, 2017). Aortic valves of women with severe AS show less AVC on CT when compared to men with similar hemodynamic severity of AS, but similar progression rates were found in males and females (Aggarwal et al., 2013; Clavel et al., 2013, 2014; Thomassen et al., 2017). Recently, it was hypothesized that more valvular fibrosis might explain the basis of this sex-related discrepancy between the AVC load and hemodynamic severity in females (Simard et al., 2017). Insight into molecular calcification processes may help to define appropriate interventions to halt or reduce progression.

Once present, AVC progresses and development of hemodynamically evident aortic valve disease is a common feature, requiring regular monitoring using echocardiography (and computed tomography). Addition of biomarkers to optimize risk assessment of progressive diseases would be useful from the initial phase onward. The ESC guidelines only integrate a possible role for NTproBNP in timing of aortic valve replacement though (Baumgartner et al., 2017). This might be due to the fact that most studies focus on the identification of biomarkers in patients with advanced aortic valve disease.

Therefore, we aimed to explore the differences in circulating biomarkers holding potential for further investigation in the early phase of AVC in a low risk population.

## Materials and Methods

### Study Population

In this cross-sectional observational study, patients without clinically overt vascular disease (other than lone atrial fibrillation) who underwent cardiac Computed Tomography (CT) (January 2008–March 2011) in the work-up for pulmonary vein isolation or general screening were screened. EDTA-plasma was available from 180 patients and these were selected for this study. Ten patients were excluded, as biomarker analysis in those patients returned a value within the limits of detection for less than 15% of the proteins, due to a technical error in the measurement. Thus, 170 patients (*n* = 48 atrial fibrillation, *n* = 122 sinus rhythm) constituted the final population for the current study. This study was approved by the Institutional Review Board.

### Computed Tomography

All patients underwent a non-contrast enhanced coronary calcium scan as described previously, performed on a Philips Brilliance 64-slice MSCT scanner (Brilliance 64; Philips Healthcare, Best, Netherlands) or a 2nd generation Dual source CT scanner (Siemens Somatom Definition Flash 2^∗^128-slice, Siemens Healthineers, Forchheim, Germany) (Weijs et al., 2012). Quantitative assessment (expressed as Agatston scores) of AVC was performed by two independent observers. Presence of AVC was defined as Agatston score >0.

### Biomarkers

Proteins were quantified by real-time PCR in all EDTA-plasma samples using the Olink Proseek Multiplex Cardiovascular I kit (Olink Proteomics, Uppsala, Sweden), as described previously (Assarsson et al., 2014). Interleukin 4 (IL4), Natriuretic Peptides B (BNP), and Melusin (ITGB1BP2) were excluded from further analyses due to low call rates (valid measurement in <85% of samples). Values below the Limit of Detection (LOD) were replaced by the LOD value^1^. Data from the panels were normalized to the median of 0 for each protein, enabling comparisons between measurements from different panels. The panel provides NPX-values which allow for relative quantification comparisons for the same protein across samples.

### Statistical Analyses

Statistical analyses were performed using SPSS version 22 (IBM Corp., Armonk, NY, United States). Normally distributed continuous variables are expressed as mean ± standard deviation (SD) and compared using the independent samples *t*-test, non-normally distributed continuous variables as median (interquartile range; IQR) and compared using the Mann-Whitney *U* test. Categorical variables are expressed as absolute numbers and percentages and tested using the Fishers exact test.

Logistic regression adjusted for age, presence of AF (and sex when appropriate) was used to determine the association between biomarkers and calcification with AVC or no AVC as the outcome. Odds ratios and 95% confidence intervals (CI) were calculated and *p* <0.05 was considered significant.

## Results

### Aortic Valve Calcification on CT

AVC was present in 34 patients: 11 females, 23 males (median [IQR] Agatston scores of the total, female and male populations were 11.3 [47.6], 15.8 [69.2], and 11.2 [40.8] respectively). In general, patients with AVC were older than patients without AVC (mean age 59 ± 6 vs. 53 ± 10 years in patients with vs. without AVC, *p* < 0.001). Other baseline characteristics were not significantly different (Supplementary Table 1). A detailed description of the study population was published previously (Weijs et al., 2012).

### Biomarkers and Valvular Calcification

**FIGURE 1:**
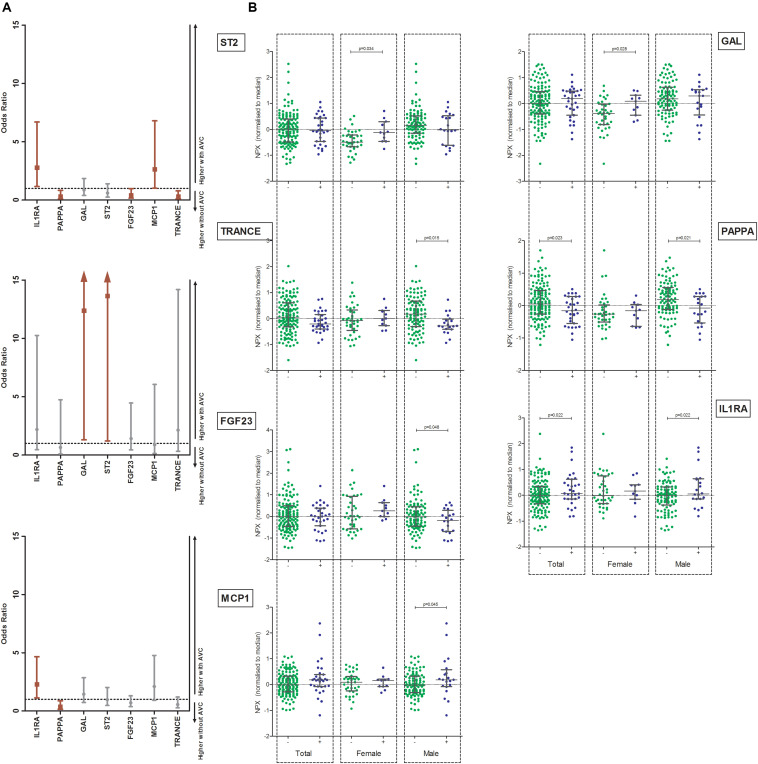
**(A)** Odds ratios and 95% confidence intervals (corrected for age, sex, and presence of AF) for biomarkers with increased or decreased odds for having AVC in the total population, female and male populations. Significant markers are displayed in red. Left panel: Total population, middle panel: female population, right panel: male population. **(B)** Unadjusted boxplots of seven biomarkers found to be significantly different between patients (male/female) with and without AVC. Median and interquartile ranges are shown. GAL, galanin peptides; MCP1, Monocyte chemotactic protein 1; PAPPA, Pappalysin-1; TRANCE, TNF-related activation induced cytokine; FGF23, Fibroblast growth factor 23; ST2, ST2-protein; IL1RA, Interleukin 1 receptor antagonist receptor.

Table 1 shows the age, sex and AF adjusted OR (and 95% CI) of all biomarkers. In the total population, Interleukin 1 receptor antagonist protein (IL1RA) was associated with increased odds of having AVC [OR 2.29 (1.13–4.65), *p* = 0.022]. Furthermore, pappalysin-1 (PAPPA) was associated with decreased odds of having AVC [OR 0.37 (0.16–0.87), *p* = 0.023] (Figure 1A).

**TABLE 1:** Odds ratios for 89 biomarkers (corrected for age, sex, and atrial fibrillation) in the total population with and without aortic valve calcification and subdivided in female and male populations.

	Total population		Female		Male	
			
	OR (95% CI)	*P*-value	OR (95% CI)	*P*-value	OR (95% CI)	*P*-value
Adrenomedullin (AM)	0.828 (0.276–2.479)	0.894	1.891 (0.204–17.499)	0.575	0.571 (0.145–2.240)	0.422
Agouti−related protein (AGRP)	0.878 (0.397–1.945)	0.749	2.179 (0.298–15.954)	0.443	0.646 (0.259–1.615)	0.350
Angiopoietin-1 receptor (TIE2)	0.906 (0.239–3.437)	0.884	29.457 (0.461–1881.605)	0.111	0.412 (0.088–1.925)	0.259
Beta-nerve growth factor (Beta-NGF)	0.775 (0.241–2.490)	0.668	1.804 (0.081–40.356)	0.710	0.642 (0.170–2.425)	0.513
Cancer antigen 125 (CA125)	0.982 (0.497–1.941)	0.959	1.097 (0.251–4.796)	0.902	1.007 (0.459–2.212)	0.986
Caspase 8 (CASP8)	0.989 (0.532–1.840)	0.973	0.836 (0.265–2.633)	0.759	1.089 (0.531–2.232)	0.816
Cathepsin D (CTSD)	1.313 (0.560–3.079)	0.532	4.289 (0.553–33.247)	0.163	1.119 (0.415–3.015)	0.824
Cathepsin L1 (CTSL1)	1.654 (0.474–5.771)	0.430	5.412 (0.142–206.068)	0.363	1.322 (0.340–5.147)	0.687
C-C motif chemokine 3 (CCL3)	1.528 (0.501–4.660)	0.456	0.786 (0.090–6.881)	0.827	1.651 (0.430–6.340)	0.466
C-C motif chemokine 4 (CCL4)	1.539 (0.850–2.786)	0.154	1.033 (0.392–2.725)	0.948	1.655 (0.781–3.510)	0.189
C-C motif chemokine 20 (CCL20)	1.225 (0.846–1.774)	0.283	1.472 (0.546–3.965)	0.444	1.256 (0.839–1.880)	0.268
CD40 ligand (CD40L)	0.705 (0.422–1.180)	0.183	0.971 (0.349–2.699)	0.955	0.659 (0.363–1.194)	0.169
CD40L receptor (CD40)	0.599 (0.224–1.603)	0.307	1.849 (0.187–18.290)	0.599	0.400 (0.121–1.330)	0.135
Chitinase−3−like protein 1 (CHI3L1)	1.241 (0.751–2.050)	0.399	1.134 (0.411–3.128)	0.808	1.302 (0.719–2.356)	0.383
C-X−C motif chemokine 1 (CXCL1)	0.629 (0.374–1.058)	0.080	0.688 (0.233–2.032)	0.499	0.660 (0.369–1.180)	0.161
C-X−C motif chemokine 6 (CXCL6)	0.868 (0.503–1.498)	0.610	1.040 (0.494–2.190)	0.917	0.555 (0.227–1.356)	0.196
C-X−C motif chemokine 16 (CXCL16)	1.416 (0.401–4.999)	0.589	10.291 (0.424–249.714)	0.152	0.876 (0.204–3.755)	0.859
Cystatin B (CSTB)	1.194 (0.650–2.193)	0.567	1.195 (0.380–3.761)	0.760	1.315 (0.627–2.760)	0.468
Dickkopf−related protein 1 (DKK1)	0.642 (0.315–1.311)	0.224	0.773 (0.199–3.010)	0.711	0.663 (0.284–1.547)	0.341
Endothelial cell−specific molecule 1 (ESM1)	0.696 (0.280–1.733)	0.436	1.203 (0.232–6.235)	0.826	0.508 (0.154–1.673)	0.266
Eosinophil cationic protein (ECP)	1.060 (0.544–2.066)	0.864	0.280 (0.051–1.524)	0.141	1.615 (0.762–3.425)	0.211
Epidermal growth factor (EGF)	0.662 (0.425–1.032)	0.069	0.696 (0.284–1.705)	0.428	0.669 (0.403–1.111)	0.120
E-selectin (SELE)	0.978 (0.490–1.951)	0.949	1.339 (0.288–6.236)	0.710	1.051 (0.462–2.389)	0.906
Fatty acid−binding protein 4 (FABP4)	0.894 (0.373–2.142)	0.801	1.377 (0.142–13.348)	0.783	0.799 (0.291–2.193)	0.663
Fibroblast growth factor 23 (FGF23)	0.699 (0.376–1.297)	0.256	1.417 (0.451–4.456)	0.551	0.410 (0.170–0.991)	**0.048**
Follistatin (FS)	0.835 (0.354–1.968)	0.835	0.790 (0.163–3.823)	0.769	0.970 (0.329–2.859)	0.955
Fractalkine (CX3CL1)	1.148 (0.397–3.319)	0.798	2.823 (0.349–22.850)	0.331	0.559 (0.150–2.090)	0.388
Galanin peptides (GAL)	1.437 (0.723–2.853)	0.301	12.381 (1.314–116.694)	**0.028**	0.867 (0.405–1.859)	0.715
Galectin 3 (GAL3)	1.000 (0.443–2.255)	> 0.999	1.276 (0.321–5.072)	0.729	0.825 (0.304–2.238)	0.705
Growth/differentiation factor 15 (GDF-15)	1.218 (0.543–2.729)	0.632	0.968 (0.088–10.710)	0.979	1.222 (0.515–2.900)	0.650
Growth hormone (GH)	0.976 (0.801–1.189)	0.806	1.227 (0.724–2.080)	0.447	0.908 (0.715–1.154)	0.430
Heat shock 27 kDa protein (HSP27)	0.839 (0.549–1.284)	0.419	0.940 (0.383–2.306)	0.940	0.803 (0.490–1.316)	0.384
Heparin-binding EGF−like growth factor (HB-EGF)	0.355 (0.094–1.345)	0.128	0.524 (0.039–7.092)	0.627	0.317 (0.067–1.510)	0.149
Hepatocyte growth factor (HGF)	0.942 (0.419–2.121)	0.886	0.970 (0.245–3.850)	0.966	1.339 (0.389–4.612)	0.644
Interleukin 1 receptor antagonist protein (IL1RA)	2.289 (1.126–4.651)	**0.022**	2.192 (0.469–10.245)	0.319	2.790 (1.163–6.695	**0.022**
Interleukin 6 (IL6)	1.296 (0.859–1.957)	0.217	1.315 (0.455–3.803)	0.613	1.326 (0.824–2.133)	0.245
Interleukin-6 receptor subunit alpha (IL6RA)	0.739 (0.281–1.941)	0.539	0.954 (0.126–7.239)	0.964	0.763 (0.243–2.392)	0.763
Interleukin 8 (IL8)	1.713 (0.829–3.542)	0.146	1.007 (0.321–3.161)	0.991	2.698 (1.001–7.270)	0.050
Interleukin 16 (IL16)	1.033 (0.463–2.306)	0.937	2.637 (0.233–29.828)	0.433	0.871 (0.364–2.084)	0.757
Interleukin 18 (IL18)	0.831 (0.377–1.828)	0.645	1.457 (0.282–7.529)	0.653	0.746 (0.302–1.845)	0.526
Interleukin-27 subunit alpha (IL27A)	2.216 (0.645–7.617)	0.206	2.004 (0.204–19.706)	0.551	1.986 (0.449–8.779)	0.366
Kallikrein 6 (KLK6)	1.270 (0.441–2.660)	0.658	17.093 (0.713–409.697)	0.080	0.681 (0.200–2.310)	0.537
Kallikrein 11 (hK11)	0.898 (0.329–2.453)	0.834	3.081 (0.355–26.763)	0.308	0.667 (0.208–2.141)	0.496
Lectin−like oxidized LDL receptor 1 (LOX1)	1.012 (0.513–1.995)	0.972	0.965 (0.211–4.417)	0.963	1.150 (0.530–2.499)	0.723
Leptin (LEP)	1.705 (0.992–2.929)	0.053	3.294 (0.828–13.098)	0.091	1.617 (0.853–3.068)	0.141
Macrophage colony stimulating factor (CSF1)	2.330 (0.410–13.245)	0.340	5.189 (0.141–190.728)	0.371	1.709 (0.219–13.309)	0.609
Matrix metalloproteinase 1 (MMP1)	0.863 (0.463–1.607)	0.641	0.945 (0.308–2.894)	0.920	1.002 (0.452–2.220)	0.996
Matrix metalloproteinase 3 (MMP3)	0.998 (0.500–1.990)	0.995	5.854 (0.932–36.759)	0.059	0.562 (0.218–1.446)	0.232
Matrix metalloproteinase (MMP7)	1.517 (0.687–3.349)	0.302	1.731 (0.433–6.918)	0.438	1.518 (0.564–4.089)	0.409
Matrix metalloproteinase (MMP10)	1.278 (0.651–2.510)	0.476	1.793 (0.469–6.863)	0.394	1.133 (0.492–2.607)	0.770
Matrix metalloproteinase 12 (MMP12)	1.583 (0.888–2.823)	0.119	1.121 (0.293–4.297)	0.867	1.600 (0.836–3.059)	0.156
Membrane−bound aminopeptidase P (mAmP)	1.031 (0.711–1.494)	0.874	1.220 (0.536–2.775)	0.636	0.892 (0.573–1.387)	0.612
Monocyte chemotactic protein 1 (MCP1)	2.098 (0.922–4.772)	0.077	0.880 (0.128–6.069)	0.897	2.635 (1.021–6.805)	**0.045**
Myeloperoxidase (MPO)	1.011 (0.268–3.821)	0.987	1.535 (0.064–37.027)	0.792	0.784 (0.165–3.723)	0.760
Myoglobin (MB)	1.387 (0.688–2.793)	0.360	2.616 (0.466–14.682)	0.274	1.009 (0.445–2.288)	0.983
NF−kappa−B essential modulator (NEMO)	0.664 (0.365–1.206)	0.179	0.562 (0.146–2.166)	0.403	0.722 (0.377–1.384)	0.327
N−terminal pro −B−type natriuretic peptide (NT-proBNP)	0.938 (0.617–1.426)	0.766	0.976 (0.356–2.680)	0.963	0.841 (0.521–1.356)	0.477
Osteoprotegerin (OPG)	1.449 (0.500–4.197)	0.495	2.777 (0.384–20.076)	0.311	1.246 (0.320–4.857)	0.751
Pappalysin−1 (PAPPA)	0.367 (0.155–0.870)	**0.023**	0.644 (0.088–4.738)	0.666	0.303 (0.110–0.836)	**0.021**
Pentraxin-related protein PTX3 (PTX3)	0.564 (0.234–1.358)	0.201	0.878 (0.164–4.702)	0.880	0.506 (0.172–1.485)	0.215
Placenta growth factor (PIGF)	0.849 (0.252–2.861)	0.792	4.359 (0.292–65.031)	0.286	0.480 (0.108–2.127)	0.334
Platelet-derived growth factor subunit B (PDGFsuB)	0.813 (0.577–1.152)	0.246	0.582 (0.275–1.234)	0.158	0.937 (0.635–1.382)	0.742
Platelet endothelial cell adhesion molecule (PECAM1)	0.600 (0.225–1.602)	0.308	4.398 (0.300–64.463)	0.280	0.393 (0.125–1.238)	0.111
Prolactin (PRL)	1.031 (0.586–1.812)	0.916	1.796 (0.577–5.594)	0.312	0.705 (0.333–1.493)	0.361
Proteinase-activated receptor 1 (PAR1)	0.733 (0.243–2.208)	0.580	1.501 (0.152–14.851)	0.729	0.638 (0.179–2.269)	0.487
Protein S100-A12 (EN-RAGE)	1.179 (0.627–2.216)	0.609	1.509 (0.470–4.847)	0.489	1.088 (0.497–2.380)	0.832
Proto-oncogene tyrosine−protein kinase Src (SRC)	0.705 (0.418–1.186)	0.188	0.962 (0.350–2.641)	0.939	0.650 (0.347–1.218)	0.178
P-selectin glycoprotein ligand 1 (PSGL-1)	1.749 (0.222–13.749)	0.595	62.819 (0.284–13874.08)	0.133	0.670 (0.060–7.443)	0.744
Receptor for advanced glycosylation end products (RAGE)	0696 (0.260–1.863)	0.470	7.234 (0.529–98.950)	0.138	0.306 (0.087–1.082)	0.066
Renin (REN)	1.438 (0.816–2.533)	0.209	2.332 (0.696–7.819)	0.170	1.073 (0.527–2.181)	0.847
Resistin (RETN)	0.704 (0.335–1.480)	0.354	1.503 (0.231–9.780)	0.670	0.651 (0.276–1.534)	0.326
SIR2-like protein (SIRT2)	0.908 (0.674–1.224)	0.526	0.856 (0.533–1.375)	0.521	0.960 (0.654–1.408)	0.834
Spondin 1 (SPON1)	0.515 (0.154–1.724)	0.282	1.677 (0.050–56.731)	0.773	0.435 (0.114–1.657)	0.222
ST2 protein (ST2)	0.975 (0.473–2.012)	0.946	13.638 (1.211–153.533)	**0.034**	0.604 (0.258–1.411)	0.244
Stem cell factor (SCF)	0.822 (0.272–2.488)	0.729	2.421 (0.182–32.147)	0.503	0.566 (0.158–2.023)	0.381
T-cell immunoglobulin and mucin domain 1 (TIM)	1.448 (0.784–2.677)	0.237	1.152 (0.296–4.484)	0.838	1.548 (0.766–3.129)	0.223
Thrombomodulin (TM)	1.361 (0.396–4.684)	0.624	269.71 (3.057–23798.388)	**0.014**	0.497 (0.115–2.149)	0.349
Tissue factor (TF)	1.219 (0.346–4.297)	0.758	6.166 (0.308–123.627)	0.234	0.752 (0.174–3.246)	0.702
Tissue-type plasminogen activator (tPA)	0.612 (0.284–1.322)	0.211	0.683 (0.099–4.715)	0.699	0.581 (0.248–1.360)	0.211
TNF-related activation−induced cytokine (TRANCE)	0.560 (0.262–1.198)	0.135	2.143 (0.324–14.187)	0.429	0.313 (0.123–0.799)	**0.015**
TNF-related apoptosis-inducing ligand (TRAIL)	1.554 (0.379–6.375)	0.541	4.111 (0.247–68.385)	0.324	1.120 (0.214–5.866)	0.893
TNF-related apoptosis-inducing ligand receptor 2 (TRAILR2)	1.185 (0.349–4.021)	0.786	2.140 (0.108–42.467)	0.618	1.128 (0.276–4.611)	0.867
Tumor necrosis factor receptor superfamily member 6 (FAS)	0.906 (0.297–2.766)	0.862	6.474 (0.575–72.853)	0.130	0.475 (0.120–1.879)	0.289
Tumor necrosis factor ligand superfamily member 14 (TNFSF14)	2.401 (0.840–6.860)	0.102	3.224 (0.327–31.749)	0.316	2.254 (0.699–7.271)	0.174
Tumor necrosis factor receptor 1 (TNFR1)	1.419 (0.407–4.944)	0.582	4.016 (0.304–52.991)	0.291	1.023 (0.226–4.634)	0.977
Tumor necrosis factor receptor 2 (TNFR2)	1.401 (0.546–3.592)	0.483	3.164 (0.324–30.891)	0.322	1.209 (0.412–3.545)	0.729
Urokinase plasminogen activator surface receptor (UPAR)	3.070 (0.879–10.726)	0.079	4.499 (0.267–75.939)	0.297	3.195 (0.699–14.610)	0.134
Vascular endothelial growth factor A (VEGF-A)	1.094 (0.309–3.870)	0.889	5.119 (0.293–89.384)	0.263	0.785 (0.182–3.382)	0.745
Vascular endothelial growth factor D (VEGF-D)	1.093 (0.478–2.499)	0.833	2.614 (0.364–18.754)	0.339	0.957 (0.451–2.032)	0.909

*Significant values are indicated in bold.*

The abovementioned differences of IL1RA and PAPPA were driven by the male population [IL1RA: OR 2.79 (1.16–6.70), *p* = 0.022 and PAPPA: OR 0.30 (0.11–0.84), *p* = 0.021 respectively]. Furthermore, TRANCE and fibroblast growth factor 23 (FGF23) were lower and monocyte chemotactic protein 1 (MCP1) was higher in males with AVC than without AVC [TRANCE: OR 0.32 (0.12–0.80), *p* = 0.015; FGF23: OR 0.41 (0.45–2.29), *p* = 0.048 and MCP1: OR 2.64 (1.02–6.81), *p* = 0.045] (Figure 1).

In the female population, galanin peptides (GAL) and ST2 protein (ST2) odds ratios were higher in females with AVC than in females without AVC [GAL: OR 12.38 (1.31–116.69), *p* = 0.028; ST2: OR 13.64 (1.21–153.33), *p* = 0.034] (Figure 1A).

Distributions of biomarkers significantly associated with AVC are shown in Figure 1B.

## Discussion and Conclusion

In this study we show differential expression differences in seven circulating biomarkers that might be associated with AVC in early stage aortic valve calcification. These biomarkers are involved in all three processes relevant for aortic valve degeneration, namely inflammation, fibrosis and calcification. Moreover, we report higher expression of fibrosis markers in the early phase of AVC in females, whereas higher expression of calcification and inflammatory markers were found in males.

The progressive character of aortic stenosis and the absence of a medication-based treatment triggered cardiovascular research to identify more precise mechanisms underlying the initiation of AVC and interactions between different pathways (Rajamannan et al., 2003; Clark-Greuel et al., 2007; Furukawa, 2014; Pawade et al., 2015; Peeters et al., 2018). Studies investigating in a follow-up design the potential role of circulating biomarkers in aortic stenosis are scarce and current guidelines only recommend repeated measurements of markedly elevated natriuretic peptides. Whilst these are incorporated in the most recent guidelines, their actual role in clinical management is not clearly defined (Baumgartner et al., 2017). Emerging studies investigate the potential utility of other biomarkers, such as troponin-T, troponin-I, ST2, growth differentiation factor-15 (GDF-15) and galectin-3 (Rosjo et al., 2011; Chin et al., 2014; Krau et al., 2015; Lancellotti et al., 2015; Arangalage et al., 2016; Henri et al., 2016; Shen et al., 2018). A recent study investigating multiple biomarkers of cardiovascular stress revealed that a combination of GDF-15, sST2, and NT-proBNP provided prognostic implications in patients with AS, and with that, a net improvement in risk stratification for mortality after both conventional aortic valve replacement and TAVI (Lindman et al., 2015). Therefore, multiple biomarkers reflecting various disease mechanisms will be useful in diagnosing aortic valve disease progression.

Recently, it was proposed that in aortic valve disease, sex-specific mechanisms should be investigated in future studies (Sritharen et al., 2017). Women who develop severe aortic valve disease have a lower valvular calcium content when compared to men (Aggarwal et al., 2013), suggesting a more dominant role for fibrosis in disease progression in women. The effects of estrogen and testosterone are thought to play a role in determination of the dominance of fibrosis in women vs. the calcification dominance in men (Sritharen et al., 2017). Therefore, we used a multiple biomarker approach to reflect disease mechanisms, and in our study, we confirm a higher expression of ST2 (myocyte stress and fibrosis; Shah and Januzzi, 2010; Lindman et al., 2015; Chin et al., 2016) and galanin peptides (myocardial remodeling in response to stress; Timotin et al., 2017) in association with AVC in women. In men, aortic valve disease is considered to be dominated by calcification, and in the current study lower expression levels of TRANCE (or RANKL) were associated with AVC. TRANCE has been shown to promote matrix calcification by inducing expression of osteoblast-associated genes, indicating a transition toward an osteogenic environment (Kaden et al., 2004). However, in our study we investigate the early stages of AVC, indicated by the low Agatston scores present in our patient population. Also lower expression of pappalysin-1, involved in insulin-like growth factor-1 signaling and osteoblast differentiation of valvular interstitial cells (VICs) (Choi et al., 2017), was associated with AVC in men. Additionally, lower expression of FGF23, a phosphatonin regulating phosphate levels involved in metabolic bone disease and vascular calcification, was associated with AVC in men. These data suggest that triggers for VIC phenotype change differ between phases of aortic valve disease progression. Moreover, circulating biomarkers involved in inflammation, oxidative stress and endothelial activation (IL1RA, MCP-1) (Deshmane et al., 2009; Herder et al., 2017) showed higher expression which is in line with previous reports that inflammation and oxidative stress relates to increased calcification (Aikawa et al., 2007). Additionally, our data confirm that AVC is actively regulated involving cellular and humoral factors that may offer targets for diagnosis and intervention. The results of the current study show new insights in biomarkers involved in aortic valve disease in a low risk population without significant risk factors for AVC. Therefore, our study adds valuable information to increase knowledge on the mechanisms of aortic valve disease. However, cautious interpretation is warranted. This retrospective, cross-sectional study with an explorative nature has a relatively small sample size, especially when stratified by sex. Furthermore, the size of this study does not allow for multiple corrections (for instance for aspirin, which possibly is associated with fibrosis). Therefore, the biomarker panel results need to be confirmed in larger studies.

Genesis and progression of aortic valve disease is a complex process. We found a number of biomarkers involved in several processes associated with aortic valve disease. Single biomarkers clearly lack sensitivity to form the base for analyzing all processes involved at different stages (including the initiation phase) of the disease, given that these biomarkers might be derived from different sources within the body. Investigating panels of biomarkers in future studies can overcome this problem in addition to further development of imaging technologies to visualize the disease in its earliest/premature phases. Moreover, integration of (a combination of) specific biomarkers and imaging could more successfully assess the risk of rapid progression, which would facilitate patient counseling and help personalize follow up of patients. Ultimately, gaining knowledge in the processes involved in the genesis and the progression phases of aortic valve disease will provide us with opportunities to investigate potential therapeutic targets to slow/reduce/regress AVC and disease progression. With that, the opportunity to delay surgical interventions in patients with aortic valve disease might be imminent.

## Disclosure

JW reports grants from Institutional grants – Agfa, Bayer, GE, Optimed, Philips, Siemens, personal fees from Speaker’s bureau – Bayer, Siemens, outside the submitted work. LF reports funding from MRC, BHF, DFG, EU, and Gilead.

## Data Availability Statement

This article contains previously unpublished data. The name of the repository and accession number(s) are not available.

## Ethics Statement

This study was approved by the Institutional Review Board of the Maastricht University Medical Center. The patients/participants provided their written informed consent to participate in this study.

## Author Contributions

FP, ED, BW, LF, WC, and PK conducted the literature search, analyzed the data, and created the figures. FP, BK, JW, SM, HC, ED, and LS wrote the manuscript. FP, BK, SM, HC, ED, and LS conceived and designed the study. LF, WC, JW, SM, BK, and PK acquired and interpreted the data. HC and LS supervised the study. All authors contributed to the article and approved the submitted version.

## Conflict of Interest

The authors declare that the research was conducted in the absence of any commercial or financial relationships that could be construed as a potential conflict of interest.

## Footnotes

1 http://www.olink.com/data-you-can-trust/validation/
