# Supplementary material for: Biomarkers Associated With Aortic Valve Calcification: Should We Focus on Sex Specific Processes?
Source: Front Cell Dev Biol. 2020 Jul 10;8:604. doi: 10.3389/fcell.2020.00604 (PMC7366171; doi:10.3389/fcell.2020.00604)
Supplement: Supplementary file 1 [file Table_1.DOCX]

Supplemental table 1: baseline characteristics

|  | Total population (n=170) | | | Female (n=49) | | | Male (n=121) | | |
| --- | --- | --- | --- | --- | --- | --- | --- | --- | --- |
|  | AVC- (n=136) | AVC+  (n=34) | p-value (95% CI) | AVC-  (n=38) | AVC+ (n=11) | p-value | AVC-  (n=98) | AVC+  (n=23) | p-value |
| Demographics | | | | | | | | | |
| Age (years) | 52.8 ± 9.6 | 59.0 ± 6.0 | <0.001  (-8.92;-3.64) | 54.5 ± 9.8 | 61.4 ± 4.1 | 0.001  (-10.89;-2.78) | 52.1 ± 9.5 | 57.9 ± 6.6 | 0.006  (-10.00;-1.70) |
| Sex (Female) | 38 (27.9) | 11 (32.4) | 0.673 | N/A | N/A | N/A | N/A | N/A | N/A |
| Systolic blood pressure (mmHg) | 127.0 ± 10.9 | 125.2 ± 8.2 | 0.364  (-2.13;5.78) | 125.9 ± 12.6 | 124.4 ± 7.2 | 0.703  (-6.49;9.55) | 127.5 ± 10.2 | 125.6 ± 8.8 | 0.423  (-2.72;6.44) |
| Diastolic blood pressure (mmHg) | 79.6 ± 8.2 | 79.6 ± 9.9 | 0.136  (-6.82;0.94) | 76.5 ± 11.4 | 80.6 ± 15.3 | 0.328  (-12.71;4.33) | 80.8 ± 9.0 | 83.4 ± 9.6 | 0.215  (-6.81;1.55) |
| BMI (kg/m^2^) | 25.6 ± 2.9 | 26.2 ± 2.5 | 0.358  (-1.74;0.63) | 24.4 ± 2.9 | 25.0 ± 2.1 | 0.655  (-2.95;1.88) | 26.1 ± 2.7 | 26.5 ± 2.6 | 0.470  (-1.82;0.85) |
| BSA (m^2^) | 2.0 ± 0.2 | 1.9 ± 0.2 | 0.578  (-0.08;0.13 | 1.8 ± 0.1 | 1.8 ± 0.1 | 0.861  (-0.13;0.11) | 2.1 ± 0.2 | 2.0 ± 0.1 | 0.223  (-0.04;0.17) |
| Atrial fibrillation | 40 (29.4) | 8 (23.5) | 0.670 | 15 (39.5) | 1 (9.1) | 0.076 | 25 (25.5) | 7 (30.4) | 0.609 |
| AF duration (months) | 16.5 [40] | 72.9 [151] | 0.223 | 11 [28] | N/A** | N/A | 18 [42] | 29 [124] | 0.563 |
| Medication | | | | | | | | | |
| VKA | 8 (5.9) | 4 (11.8) | 0.261 | 2 (5.3) | 1 (9.1) | 0.542 | 6 (6.1) | 3 (13.0) | 0.370 |
| Aspirin | 37 (27.8) | 10 (30.3) | 0.830 | 13 (35.1) | 0 (0.0) | 0.043 | 24 (25.0) | 10 (43.5) | 0.121 |
| Beta blocker | 30 (22.6) | 4 (12.1) | 0.233 | 7 (18.9) | 0 (0.0) | 0.318 | 23 (24.0) | 4 (17.4) | 0.590 |
| Digoxin | 5 (3.8) | 1 (3.0) | >0.999 | 3 (8.1) | 0 (0.0) | >0.999 | 2 (2.1) | 1 (4.3) | 0.478 |
| Statin | 14 (10.3) | 6 (17.6) | 0.241 | 2 (5.3) | 1 (9.1) | 0.542 | 12 (12.2) | 5 (21.7) | 0.314 |
| Lab | | | | | | | | | |
| Creatinine (umol/L) | 84.6 ± 12.6 | 87.6 ± 13.4 | 0.234  (-8.01;1.97) | 75.1 ± 10.8 | 83.2 ± 11.4 | 0.054  (-16.37;0.15) | 88.3 ± 11.2 | 89.3 ± 14.0 | 0.705  (-6.53;4.44) |
| eGFR (ml/min/1.73m^2^) | 92.7 ± 14.6 | 83.9 ± 14.3 | 0.010  (2.12;15.46) | 88.2 ± 15.9 | 81.4 ± 15.7 | 0.317  (-6.89;20.61) | 94.7 ± 13.7 | 85.0 ± 14.0 | 0.012  (2.19;17.31) |
| Total cholesterol (mmol/L) | 5.4 ± 1.0 | 5.4 ± 1.1 | 0.846  (-0.35;0.42) | 5.7 ± 0.9 | 5.9 ± 0.6 | 0.434  (-0.87;0.38) | 5.3 ± 1.0 | 5.2 ± 1.2 | 0.532  (-0.33;0.63) |
| LDL- cholesterol (mmol/L) | 3.4 ± 0.9 | 3.4 ± 1.0 | 0.476  (-0.47;0.22) | 3.4 ± 0.8 | 3.5 ± 0.8 | 0.513  (-0.72;0.36) | 3.3 ± 0.9 | 3.4 ± 1.0 | 0.692  (-0.53;0.35) |
| HDL-cholesterol (mmol/L) | 1.3 ± 0.4 | 1.3 ± 0.5 | 0.502  (-0.19;0.10) | 1.4 ± 0.4 | 1.6 ± 0.5 | 0.317  (-0.41;0.13) | 1.2 ± 0.3 | 1.2 ± 0.3 | 0.866  (-0.15;0.17) |
| Triglycerides (mmol/L) | 1.6 ± 0.9 | 1.5 ± 0.8 | 0.560  (-0.24;0.44) | 1.6 ± 0.8 | 1.6 ± 1.1 | 0.824  (-0.92;0.75) | 1.7 ± 1.0 | 1.4 ± 0.6 | 0.360  (-0.22;0.61) |
| Glucose (mmol/L) | 5.5 ± 0.7 | 5.6 ± 0.7 | 0.6420  (-0.37;0.23) | 5.5 ± 0.7 | 5.3 ± 0.9 | 0.619  (-0.43;0.72) | 5.5 ± 0.7 | 5.7 ± 0.7 | 0.349  (-0.53;0.19) |
| Echocardiography | | | | | | | | | |
| Left atrial diameter (mm) | 37.1 ± 4.8 | 38.8 ± 4.9 | 0.111  (-3.79;0.39) | 34.6 ± 4.5 | 35.0 ± 3.8 | 0.839  (-4.37;3.57) | 38.0 ± 4.5 | 40.0 ± 4.6 | 0.095  (-4.27;0.35) |
| IVSd (mm) | 8.6 ± 1.0 | 8.8 ± 1.0 | 0.540  (-0.58;0.31) | 8.3 ± 1.0 | 7.8 ± 0.8 | 0.300  (-0.43;1.34) | 8.8 ± 1.0 | 9.1 ± 0.9 | 0.231  (-0.80;0.20) |
| LVPWd (mm) | 8.5 ± 1.0 | 8.8 ± 0.8 | 0.09  (-0.76;0.56) | 8.3 ± 0.9 | 8.0 ± 0.6 | 0.506  (-0.52;1.04) | 8.6 ± 1.0 | 9.1 ± 0.7 | 0.007  (-0.91;-0.15) |
| EDV (ml) | 115.0 ± 25.3 | 112.3 ± 26.5 | 0.741  (-10.57;14.82) | 99.3 ± 21.8 | 94.0 ± 5.2 | 0.473  (-7.52;15.44) | 121.1 ± 24.0 | 115.5 ± 27.5 | 0.417  (-7.97;19.04) |
| ESV (ml) | 40.3 ± 13.6 | 39.2 ± 13.1 | 0.743  (-5.54;7.74) | 36.4 ± 11.3 | 33.3 ± 2.9 | 0.646  (-10.57;16.73) | 41.8 ± 14.2 | 40.2 ± 14.0 | 0.686  (-6.13;9.27) |
| LVEF (%) | 60.9 ± 4.7 | 61.7 ± 5.1 | 0.421  (-2.89;1.22) | 60.7 ± 4.3 | 61.2 ± 5.8 | 0.836  (-4.57;3.72) | 61.0 ± 4.8 | 61.9 ± 5.0 | 0.438  (-3.37;1.47) |
| CT | | | | | | | | | |
| Agatston score aortic valve | N/A | 11.3 [47.6] | N/A | N/A | 15.8 [69.2] | N/A | N/A | 11.2 [40.8] | N/A |

*Abbreviations: AVC: aortic valve calcification, BMI: Body Mass Index, BSA: Body surface area, EDV: end-diastolic volume, ESV: end-systolic volume, eGFR: estimated glomerular filtration rate, HDL: high-density lipoprotein, IVSd: interventricular septum diameter, LDL: low-density protein, LVEF: left ventricular ejection fraction, LVPWDd: left ventricular posterior wall diameter, VKA: vitamin K antagonist*
